# IgA as a potential candidate for enteric monoclonal antibody therapeutics with improved gastrointestinal stability

**DOI:** 10.1016/j.vaccine.2020.09.070

**Published:** 2020-11-03

**Authors:** Aaron L. Wallace, Matthew I. Schneider, Jacqueline R. Toomey, Ryan M. Schneider, Mark S. Klempner, Yang Wang, Lisa A. Cavacini

**Affiliations:** MassBiologics of the University of Massachusetts Medical School, 460 Walk Hill St., Mattapan, MA 02126, USA

**Keywords:** Immunoglobulin A, dIgA, sIgA, Intestinal stability

## Abstract

Mucosal surfaces of the gastrointestinal tract play an important role in immune homeostasis and defense and may be compromised by enteric disorders or infection. Therapeutic intervention using monoclonal antibody (mAb) offers the potential for treatment with minimal off-target effects as well as the possibility of limited systemic exposure when administered orally. Critically, to achieve efficacy at luminal surfaces, mAb must remain stable and functionally active in the gastrointestinal environment. To better understand the impact of isotype, class, and molecular structure on the intestinal stability of recombinant antibodies, we used an *in vitro* simulated intestinal fluid (SIF) assay to evaluate a panel of antibody candidates for enteric mAb-based therapeutics. Recombinant IgG1 was the least stable following SIF incubation, while the stability of IgA generally increased upon polymerization, with subtle differences between subclasses. Notably, patterns of variability within and between mAbs suggest that variable regions contribute to mAb stability and potentially mediate mAb susceptibility to proteases. Despite relatively rapid degradation in SIF, mAbs targeting Enterotoxigenic *Escherichia coli* (ETEC) displayed functional activity following SIF treatment, with SIgA1 showing improved function compared to SIgA2. The results of this study have implications for the design of enteric therapeutics and subsequent selection of lead candidates based upon *in vitro* intestinal stability assessments.

## 1 Introduction

Immune responses at the mucosal surface of the gastrointestinal (GI) tract play a crucial role in defense against pathogens as well as regulating inflammatory responses against commensal gut microbes and food antigens. The consequences of enteric infection or of immune dysregulation can be significant; immune disorders such as inflammatory bowel disease (IBD) affect over 0.3% of the population in parts of the developed world [1], and it is estimated that bacterial diarrheal diseases alone result in 1.3 million deaths per year [2]. Despite considerable effort, significant challenges remain in the treatment of GI infections. Vaccines against many pathogens have yet to be developed, and in any event may not be sufficient to protect some high-risk groups such as very young, elderly, or immunocompromised individuals. Thus, the development of new therapeutics and delivery modalities capable of preventing infection or inflammation at the mucosal surface remains an important goal.

Over the last several decades the number of licensed monoclonal antibody (mAb)-based therapeutics has increased substantially, with 79 mAbs approved by the US Food and Drug Administration as of December 2019, including several targeting IBD [3]. While such successes support the further development of enteric mAb-based therapeutics, the gastrointestinal environment presents significant challenges for mAb delivery and potency. MAb is susceptible to pH-dependent and protease-mediated cleavage in the GI tract as well as degradation by bacterial products in the colon, leading to loss of function [4], [5], [6]. Further, following delivery to luminal surfaces functional mAb must persist for sufficient time to achieve efficacy. Thus, primary design considerations for enteric mAb therapeutics must include formulation and optimization for protease resistance, retention in the GI tract, and sufficient functional half-life to achieve efficacy. Studies using IgG have shown limited success in this regard [5], [6], [7], [8], and other antibody classes have been investigated with the goal of enhanced stability at luminal surfaces [9], [10].

Both immunoglobulin G (IgG) and secretory IgA (sIgA) are abundant in mucosal secretions and surfaces. SIgA consists of IgA monomers joined covalently by J-chain and associated with the extracellular domains of the polymeric Ig receptor, termed secretory component (SC) [11]. IgA is expressed as two subclasses, IgA1 and IgA2, which differ in the length of the hinge region, glycosylation pattern, and positioning of several intermolecular disulfide bonds as well as structural morphology [11], [12]. In the intestine, sIgA functions to prevent infection and illness through immune exclusion of pathogens and toxins and performs other critical immune functions [13]. Additionally, sIgA is able to associate with mucosal surfaces [11], [13], [14], [15], and is more resistant to proteolysis than is IgG [11], [16]. The presence of SC also serves to mask several protease cleavage sites in Cα domains, leading to greater protease resistance compared to monomeric and dimeric IgA (the latter possessing J chain but lacking SC) [11], [12]. Further, a number of passive immunization studies support the efficacy of sIgA to protect against pathogens [17], [18], [19], [20], [21]. Thus, recombinant (S)IgA is a promising candidate for enteric therapeutics applications, and several SIgA-based constructs are currently being developed by groups utilizing CHO-cell and plant-based expression systems [22], [23].

Streamlining development of mAb-based therapeutics targeting the GI tract will require the inclusion of structural and functional stability assays as criteria for lead selection early in the discovery pipeline. In particular, such assays should be small-scale, require minimal material, be compatible with a number of downstream analytical methods, and permit high enough throughput to enable down selection prior to more costly and time-consuming *in vivo* studies. A number of recent studies have been undertaken to establish consensus *in vitro* digestion models using simulated gastric and intestinal fluid (SGF and SIF respectively) to accurately mimic *in vivo* digestion conditions [24], [25], [26]. While generally employed at larger scales for nutrition studies, these models have potential applications for early stage stability studies of enteric therapeutics. Here we have used a simulated intestinal stability assay to evaluate a panel of mAbs representing different isotypes, subclasses, and molecular structures directed against toxoid, bacterial, and viral antigens. Our results showed that mAbs expressed as IgA displayed better stability profiles than did IgG, with the greatest stability being observed for SIgA. Overall, this model has potential as a new tool for the early stage discovery of mAb-based therapeutics targeting the intestine and has implications for the development of oral delivery strategies.

## 2 Materials and methods

### 2.1 Antibodies and reagents

A total of four mAbs were used for these studies. MAb1 was isolated as an IgA1 from the peripheral blood mononuclear cells (PBMC) of a patient challenged with ETEC and is reactive against the heat labile enterotoxin B subunit. A recent study has extensively characterized the structure, glycosylation profile, and stability of this mAb [22]. MAb2 was isolated as an IgG1 using PBMCs from an HIV + patient and is reactive with gp120 [27]. The variable regions of both antibodies were subsequently sequenced and cloned in frame with human IgG1, IgA1 or IgA2(m1) constant regions using a proprietary DNA vector. Two antibodies from the panel described in [28] (mAb3 and mAb4 - both IgG1) were originally isolated from transgenic mice expressing human immunoglobulin genes (HuMab) immunized with the ETEC colonization factor antigen adhesin subunit CfaE, and variable regions were cloned to generate isotype-specific mAb as above. All antibodies used the kappa light chain, and all antibodies of a given isotype contained identical plasmid-encoded heavy and light constant regions. Recombinant dimeric IgA (dIgA) was produced using either stable cell lines (mAb1 and mAb2) or transiently transfected CHO cells (mAb3 and mAb4) with the ExpiCHO expression system (Life Technologies) expressing the heavy and light chain as well as J-chain. SIgA was produced by co-culturing dIgA-expressing cells with cells expressing secretory component. MAb was purified from supernatant over either a CaptureSelect (ThermoFisher) or Capto L (GE Healthcare) affinity column followed by size exclusion chromatography (SEC) using a HiLoad 26/600 Superdex column on a GE AKTA Pure chromatography system. Following SEC, mAb was dialyzed and concentrated in phosphate buffered saline (PBS) pH 7.2. Concentration was determined either by analytical SEC or by ELISA by comparison with a known standard and mAbs were stored at 4 °C prior to use.

Heat labile enterotoxin B subunit of *E. coli* was purchased from Sigma-Aldrich (#E8656). Recombinant gp120 and the N-terminal adhesion domains of CfaE have been described previously [28], [29] and were produced at MassBiologics for this study. ETEC strain H10407 expressing CFA/I fimbriae was obtained from the American Type Culture Collection (ATCC 35401).

### 2.2 SIF stability assay

Purified mAb was evaluated for intestinal stability using a modified assay (Fig. S1) based on a consensus adult digestion model designed to simulate gastrointestinal conditions *in vitro* [26]. SIF mimicking fasting conditions was prepared using FaSSIF/FeSSIF/FaSSGF powder (Product code FFF01) from Biorelevant according to the manufacturer’s instructions. The fasting state was specifically selected to minimize confounding variables such as the presence of exogenous food protein associated with the fed state. Briefly, to prepare the dissolution buffer, 0.344 g of sodium phosphate anhydrous (NaH_2_PO_4_) and 0.619 g of NaCl was dissolved in 100mLs H_2_O and pH adjusted to 7.0. To prepare SIF, FaSSIF/FeSSIF/FaSSGF powder (0.224 g) was dissolved in 50 mL dissolution buffer to permit optimal micelle formation, after which an additional 50 mL of buffer was added and allowed to stand for at least two hours in the dark prior to use. All SIF was used within 48 h of preparation. Thirty minutes prior to the start of the assay, porcine pancreatin (10 mg/mL, Sigma-Aldrich P1625) was added and SIF was prewarmed to 37 °C.

Prior to incubation with SIF, all antibodies were diluted to a final concentration of 500 nM in deionized H_2_O. Seven hundred microliters of SIF + pancreatin was added to 16x100mm disposable glass culture tubes containing a small magnetic stir bar on a stir plate set to low spin at 37 °C. To begin the assay, 300 μL of each antibody in H_2_O was added to each culture tube for a final mAb concentration of 150 nM. Samples (200 μL) were taken at T = 5, =30, =60, and =120 min time points and immediately placed on ice in 1.5 mL microcentrifuge tubes. Halt Protease Inhibitor Cocktail (Thermo Scientific #87786) and EDTA was added to a final concentration of 1X and 5 mM, respectively. Samples were centrifuged for ten minutes at 16,000*g* at 4 °C to pellet particulates and supernatant transferred on ice to new tubes. Aliquots were used immediately for ELISA and PAGE analysis, and remaining sample was stored at −80 °C for functional assays. Each SIF assay and subsequent analysis was performed independently at least three times (with the exception of mAb3 dIgA2, where due to limited reagent N = 2).

### 2.3 Enzyme-linked immunosorbent assay (ELISA)

SIF treated samples were evaluated for immunoreactivity using antigen-specific ELISA. For IgG and mIgA ELISA, Corning polystyrene 96-well plates (#9018) were coated overnight at 4 °C with antigen (LT, gp120, or CfaE) at 100 ng/well   followed by blocking with ELISA buffer (1X Blocker BSA [ThermoFisher #37525], 0.05% Tween-20 in PBS). SIF samples were diluted in ELISA buffer to a final concentration of 1 μg/mL based upon the initial concentration of antibody used in the assay and titrated 2-fold. Following a thirty minute incubation, plates were washed 3x with PBST (PBS + 0.05% Tween-20) and incubated with horseradish peroxidase conjugated Goat-anti-human IgG (Southern Biotech #2040-03) or Goat-anti-human IgA (Southern Biotech #2050-05) in ELISA buffer for thirty minutes. After a second wash step, plates were developed using a TMB 2-Component Microwell Peroxidase Substrate Kit (SeraCare #5120-0047) and background corrected absorbance (A_450_) was determined using a Biotech Epoch plate reader with Gen5 software. In the case of dIgA and SIgA, ELISA was carried out essentially as described above with the exception of using a monoclonal mouse anti-human J-chain antibody followed by HRP-conjugated Goat-anti-mouse IgG (Southern Biotech #1030-05) for detection.

### 2.4 Hemagglutination inhibition assay

To measure the functional activity of anti-CfaE mAb3 and mAb4, hemagglutination inhibition (HAI) assays were carried out as described previously [28]. Briefly, ETEC cultures (strain H10407) were diluted in PBS to an OD_600_ of 1.0. Human erythrocytes were washed in PBS and resuspended to a final concentration of 1.5%. One hundred microliters of antibody in PBS was added to the top wells of a 96-well U-bottom plates (Nunc Thermo Scientific) in duplicate and titrated 2-fold down the plate in 50 μL PBS followed by the addition of 50 μL ETEC and 50 μL D-mannose solution to each well. After a 10 min incubation, 50 μL of blood was added to a final volume of 200 μL/well. Plates were statically incubated for two hours at 4 °C and hemagglutination observed without magnification. Results are reported as the minimal inhibitory concentration (IC_100_).

### 2.5 Statistical analysis

All data were analyzed using GraphPad Prism 8.0, with a *p*-value of ≤0.05 being the cutoff for significance. Half maximal concentrations (½max) were determined by linear regression of log-transformed ELISA data, and defined as the concentration of SIF-incubated antibody resulting in 50% of the A_450_ of the averaged undigested controls at 1 μg/mL. Samples with ½max > 1 μg/mL were assigned a maximum in-assay value of 1.0 for purposes of analysis. A significant difference in mean ½max over all timepoints (including controls with no SIF) for each mAb was determined by one-way ANOVA and without assuming unequal variances between timepoints. The average time required to degrade 50% of each mAb (T50%) was determined by interpolation from normalized log-transformed data using a sigmoidal best fit model, and significance determined by one-way ANOVA. Similar to the ½max analysis, mAbs that failed to reach a T50% over the course of the experiment were assigned a T50% equal to the longest timepoint tested (120 min). Individual comparisons between two groups were carried out using Welch’s T-test assuming unequal variances.

## 3 Results

### 3.1 Stability in SIF is influenced by mAb isotype, subclass, and multimerization state

Along with IgM, IgG and SIgA (both A1 and A2) are the primary mediators of adaptive immunity at mucosal sites. While all antibody therapeutics licensed to date have been of the IgG isotype, sIgA-based enteric therapeutics offer the potential for higher avidity, enhanced gastrointestinal stability and half-life, decreased systemic exposure, and reduced inflammatory responses. Here we assessed four mAbs targeting bacterial toxoid, fimbrial, and viral antigens expressed as IgG and/or SIgA for gastrointestinal stability using an *in vitro* SIF assay (Fig. S1). To better compare IgG with IgA, monomeric and dimeric IgA were also evaluated for three of these mAbs. The four mAbs displayed diverse variable gene usage, with >90% identity to germline for VH domains and >94% identity for VL, and CDRH3 lengths ranging from 11 to 19 amino acid residues (Table S1).

**Fig. 1:**
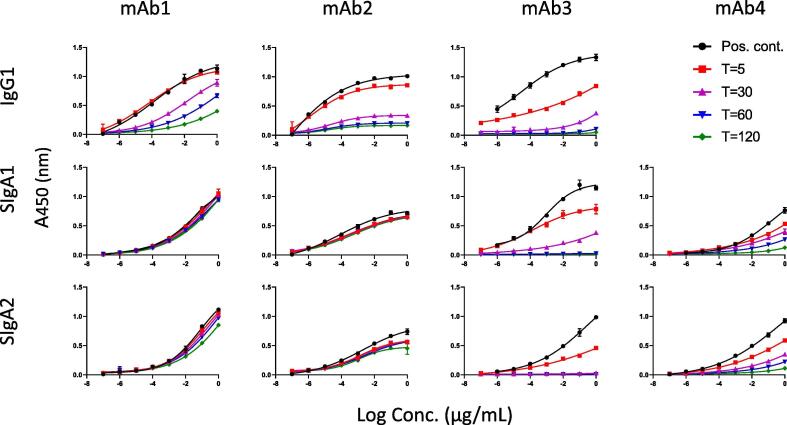
**Stability of antibodies in SIF is mediated by isotype and isomerization**. IgG and monomeric, dimeric, and secretory IgA were incubated with SIF and tested for antigen-specific binding over multiple timepoints by ELISA, with representative results for each antibody shown.

**Fig. 2:**
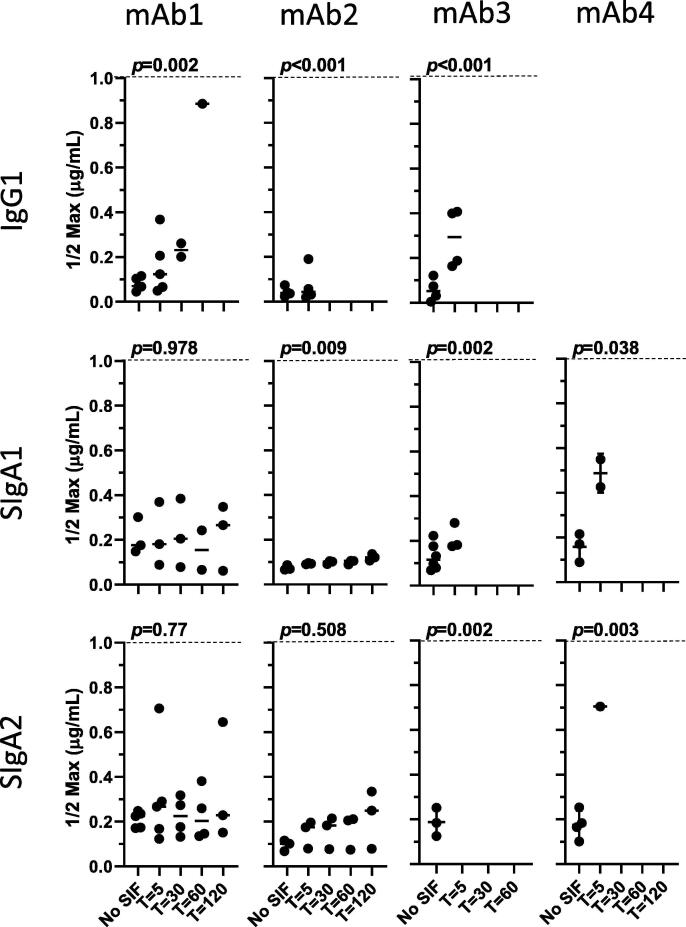
**Antibodies expressed as SIgA demonstrate improved stability in SIF compared to IgG**. SIF ½max concentrations mAbs1-3 IgG and mAbs1-4 SIgA are shown. Samples with ½ Max concentrations >1 μg/mL (shown by a dotted line) were outside of the assay limits and are not displayed, however these samples were assigned a value of 1 μg/mL for purposes of analysis.

**Fig. 3:**
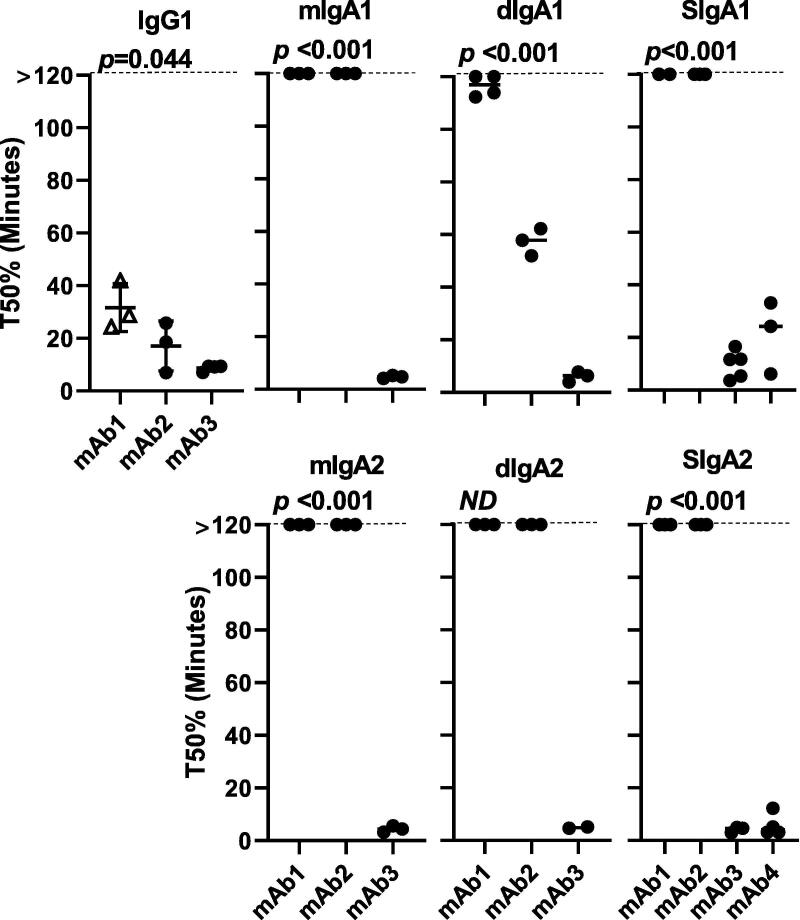
**Antibody half-life is differentially impacted by isotype and multimerization state.** Normalized T50% for each mAb was determined as the mean time in minutes for a 50% reduction in immunoreactivity relative to a positive control. Antibodies that displayed >50% immunoreactivity over the course of the assay were assigned a T50% of 120 min for purposes of analysis and are shown on the graphs for clarity. Significance across mAbs for a given construct is shown at the top of each graph. Due to the small sample size for mAb3 dIgA2 (N = 2), dIgA2 was not analyzed.

Representative antigen-specific ELISA curves across timepoints are shown in Fig. 1 (IgG and SIgA) and Fig. S2 (mIgA and dIgA). Overall, loss of signal over the course of 120 min was observed for most isotypes and subclasses evaluated. The stability of each mAb in SIF was impacted by the associated constant regions (IgG vs. IgA), molecular structure (monomer vs. dimer), and the presence or absence of SC. The immunoreactivity of IgG1 decreased over the course of the assay for mAbs1-3 (for mAb4, only SIgA was tested) as determined by increasing ½max concentrations over time, with all samples displaying ½max values outside of the upper limit of the assay (1 μg/mL) by 120 min (Fig. 2). Significant loss of immunoreactivity was also observed for mIgA1 for mAbs1-3, but was not observed for mIgA2 with the exception of mAb3 (Fig. S3). In contrast to IgG, mean ½max values for mIgA were <1 μg/mL over the course of the assay for both mAb1 and mAb2 (Fig. S3). The time required for 50% (T50%) loss of signal was also significantly shorter for IgG compared to mIgA1 for mAb1 (31.8 compared to >120 min, *p* = 0.004) and mAb2 (16.8 compared to >120 min, *p* = 0.003) (Fig. 3). Finally, the percent immunoreactivity remaining after 120 min in SIF was substantially higher for mIgA1 and mIgA2 as compared to IgG for both mAb1 and mAb2 (Table 1). These data demonstrate the enhanced stability of mIgA, particularly mIgA2, compared to IgG in our assay.

**Table 1:** **Average (mean) normalized percent immunoreactivity remaining after 120 min for all mAbs**.

	mAb1	mAb2	mAb3	mAb4
IgG	13.9	18.4	6.3	
mIgA1	80.1	49.4	1.1	
mIgA2	89.5	75.8	2.0	
dIgA1	56.8	29.2	1.5	
dIgA2	72.2	55.0	1.2	
SIgA1	93.2	78.6	1.7	19.6
SIgA2	73.8	71.7	1.2	6.6

As the majority of mucosal IgA is in the form of dimer and higher order multimers, we evaluated the effect of dimerization on the stability of recombinant IgA. Significant loss of immunoreactivity over time was observed for dIgA1 as determined by ½max concentrations (Fig. S3), and a trend towards loss of reactivity was observed for mAb1 and mAb2 dIgA2. In the case of dIgA2, a small sample size (N = 2) for mAb3 precluded a full analysis. Comparison of monomers to dimers showed a significant difference in T50% between mAb2 mIgA1 and dIgA1 (*p* = 0.002), but not between other monomer and dimer pairs (Fig. 3). While such differences may be present, they were not apparent over the two hour time course that we examined. Overall, our results suggest that dimerization did not significantly improve stability in our assay, and indeed suggest that dIgA may be less stable than monomeric forms in some cases, though further investigations need to be carried out to evaluate this.

The addition of SC can enhance proteolytic stability of dIgA as well as increase retention time at mucosal surfaces by facilitating association with the mucosal layer. Consistent with this protective role for SC, a significant loss of immunoreactivity over time for mAb1 and mAb2 was observed for only one SIgA (mAb2, SIgA1), which nonetheless had a ½max of 0.22 μg/mL at the 120 min time point (Fig. 2). Compared to IgG1, mAb1 and mAb2 SIgA1 and SIgA2 displayed lower ½max concentrations over the course of the assay, significantly increased T50%s (*p* = 0.004 for mAb1 and *p* = 0.004 for mAb2), and retained 4–5-fold greater immunoreactivity at the end of the assay (Table 1, Fig. 2, Fig. 3). Further, mAb1 and mAb2 SIgA had low ½max concentrations over all time points, while those of dIgA exceeded the upper limit of detection at later time points for some replicates (Figs. 2 and S3). Although assay limitations prevented significance comparisons of most mAb1 and mAb2 dIgA/SIgA pairs (due to T50% values being >120 min), a significant difference was observed between mAb2 dIgA1 and SIgA1 T50% (*p* = 0.002). No significant differences were observed between secretory IgA1 and IgA2 for either mAb1 or mAb2. Taken together, these data demonstrate significantly enhanced stability of SIgA compared to IgG and mIgA/dIgA in SIF for some mAbs, and offer support for the development and evaluation of SIgA-based enteric therapeutics.

Unlike the patterns observed for mAb1 and mAb2, analysis of mAb3 and mAb4 showed substantial losses of immunoreactivity over time regardless of isotype and subclass tested (Table 1, Fig. 1, Fig. 2, Fig. 3). No large isotype- or subclass- associated differences in stability were observed for mAb3, with T50%s ranging from 3.6 to 9.9 min (Fig. 3) and most forms being completely degraded by 120 min regardless of isotype, molecular structure, or the presence of SC (Table 1). Comparisons of mAb4 SIgA1 and SIgA2 showed that SIgA1 had the better stability profile overall (mean T50% = 36.3 min), with 19.6% of signal remaining after 120 min (Table 1). Overall, our results show that antibody isotype, multimerization state, and the addition of secretory component can all impact the stability profiles of mAbs.

It was notable that although the four mAbs used in this study were cloned into the same vectors and had identical Fc regions and structure, they nevertheless differed in their stability in SIF as determined by ELISA. Significant differences in T50% values were observed between the different mAbs expressed as IgG1 as well as monomeric, dimeric, and secretory IgA. In the case of IgG1, all four mAbs displayed rapid loss of immunoreactivity (Fig. 3), while for other molecular forms mAb1 and mAb2 displayed better stability profiles than did mAb3 and (for SIgA) mAb4. Interestingly, T50% comparison between mAb1 and mAb2 showed that both were quite stable (T50% >120 min) when expressed as mIgA1 but displayed different stability profiles when expressed as dIgA1, (Fig. 2, Fig. 3, S3 and Table 1). These data suggest the possibility that the variable regions, as well as isotype and structure, may contribute directly and/or indirectly to the stability profile of a given mAb in some instances.

### 3.2 Antibody stability in SIF is associated with functional differences

The potential for high avidity and enhanced GI stability of sIgA relative to IgG and mIgA has generated interest in its potential for therapeutic use as a recombinant therapeutic. To investigate the effects of incubation with SIF on the functional activity of SIgA, we examined the ability of the anti-ETEC antibody mAb3 expressed as SIgA1 and SIgA2 to inhibit bacterial-mediated hemagglutination and compared it to SIgA from a second anti-ETEC mAb (mAb4). The minimal inhibitory concentration (IC_100_) is reported as the averaged results of two independent and blinded experiments in Table 2. While SIF by itself had no detectable effect (top row), the lower IC_100_ observed for mAb3 SIgA1 and SIgA2 and mAb4 SIgA1 at several time points compared to the positive control (no SIF) may indicate that SIF did impact the assay to some extent, though this does not affect comparisons between the different SIF-treated mAbs. Of the four SIgAs tested, mAb4 SIgA2 was the least functionally stable with IC_100_ above the detection limit of the assay (>1.25 μg/mL) for all SIF-treated time points. MAb3 SIgA2 was more stable, with a detectable IC_100_ of 0.16 μg/mL at five minutes, followed by mAb3 SIgA1 which had a detectable IC_100_ up to the 30 min time point. MAb4 SIgA1 proved to be the most stable, with an IC_100_ of 1.25 μg/mL (the upper limit of detection for this assay) even after two hours. These results were in agreement with ELISA data, which showed similar patterns between these SIgAs, and demonstrate that stability profiles in binding assays correlate with functional differences. While SIgA1 was more functionally stable than SIgA2 in this assay, further evaluations are clearly needed prior to any conclusion regarding the overall functional stability of mAb expressed as SIgA1 compared to SIgA2.

**Table 2:** **Effects of SIF incubation on antibody function evaluated by Hemagglutination Inhibition Assay**. The anti-ETEC mAbs 3 and 4 were incubated in SIF, and samples from multiple time points evaluated for their ability to inhibit hemagglutination. Each assay was performed twice and the averaged result is reported as the minimal concentration at which inhibition was observed (IC_100_ in μg/mL). The highest concentration tested for inhibition was 1.25 μg/mL.

	**mAb3**		**mAb4**	
	SIgA1	SIgA2	SIgA1	SIgA2
**SIF control**	>1.25	>1.25	>1.25	>1.25
**No SIF**	0.64	0.94	0.94	0.94
**T = 5**	0.10	0.16	0.16	>1.25
**T = 30**	0.39	>1.25	0.31	>1.25
**T = 60**	>1.25	>1.25	0.63	>1.25
**T = 120**	>1.25	>1.25	1.25	>1.25

## 4 Discussion

Antibody Fc domains link effector functions to antigen binding by variable regions, contribute to antibody polymerization (dIgA and sIgA), and in the case of the Fc-associated secretory component of sIgA, links adaptive and innate immune responses, facilitates the association of the antibody with the intestinal mucosa, and can be required for protection [30]. Resistance to GI-mediated cleavage and retention of antibody binding capacity in the intestine are therefore critical considerations for therapeutics development. To date a number of simulated digestion models have been proposed, and efforts have focused on incorporating all available information across multiple studies regarding digestion parameters to develop a next generation of standardized models [24], [26], [31]. One goal of the current study was to use this information to allow for high-throughput, small scale *in vitro* assessments of intestinal stability profiles to facilitate early stage discovery of enteric therapeutics prior to the use of more costly and time-consuming *in vivo* studies. Given the functional benefits conferred by the association of variable (VH/VL) and constant (Fc) regions, we focused on the stability of intact mAb rather than the analysis of degradation products. Our assay was designed to simulate the adult intestinal fasting state, as it represents a harsher intestinal environment than that of the adult or infant fed states [25], [26], [32], however it can be easily modified as needed to reflect a range of fed or fasting conditions and could be added downstream to an *in vitro* simulated gastric fluid (SGF) assay to generate a sequential SGF/SIF model. The current SIF assay is especially advantageous for studies evaluating the stability, functionality, and retention time in the GI tract of oral antibody-based therapeutics. As formulations for protection of orally delivered antibody therapeutics from gastric digestion and to facilitate release at luminal sites may range from encapsulation strategies to the use of mAbs as a food additive or mixed into infant formula [4], [9], [21], there will be an ongoing need to incorporate stability studies such as that proposed here to expedite discovery pipelines and advance lead candidates to *in vivo* studies.

To date, all full length antibodies approved for clinical use by the EMA and FDA have been IgG. IgG comprises approximately 70–85% of serum antibody, mediates a variety of effector functions, and possesses a long serum half-life, making this an optimal isotype for many therapeutic mAbs [33], [34]. IgA levels in serum are low (≈15%, of which ≈90% is IgA1), but IgA is the dominant isotype at mucosal surfaces in the lumen where it plays a critical role in defense against pathogens and in mediating immune responses to commensal bacteria, primarily in the form of sIgA [11], [14]. In the present study, IgG displayed the least favorable stability profile compared to other monomers or dimeric forms. IgG is more susceptible to cleavage by trypsin, chymotrypsin, and duodenal fluid than is IgA, though Fab cleaved from IgG can still bind antigen [35], and the loss of immunoreactivity observed by ELISA was likely due primarily to the cleavage of the Fc region, which would impact detection. Nonetheless, Fab are susceptible to rapid clearance from luminal surfaces due to peristalsis, which may limit their window of efficacy as compared to intact mAb. Particularly in the case of mAb1 and mAb2, IgA was more stable than IgG1, while differences between all isotypes and subclasses tested were less pronounced for mAb3 and mAb4. Our observations here are in agreement with recently published stability tests in simulated gastric fluid using mAb1, which also found enhanced stability of SIgA compared to IgG1 for this antibody [22].

IgA1 is more susceptible to cleavage by a number of bacterially secreted proteases than is IgA2, primarily due to the extended hinge region of the former [11], [12], [36]. Consistent with these observations, we observed that mIgA2 was somewhat more stable than mIgA1 in SIF (Fig. S3, Table 1, Table 2). Surprisingly, dimeric IgAs were less stable than their corresponding monomers over the 120 min incubation in SIF as determined by ELISA. Although the reason is unclear, we note that dIgA was detected using an anti-J-chain mAb, whereas mIgA was detected using commercial HRP-conjugated polyclonal IgA. Thus, cleavage of the α-tailpiece or elsewhere in the constant region could completely abrogate detection of dIgA, while mIgA might be less affected by such cleavages when analyzed by ELISA. Further studies will need to be carried out to fully characterize the relative intestinal stability of these two IgA subclasses.

Secretory IgA (either dimeric or higher-order multimers) is the dominant form of IgA in the mucosa, and has been shown to be more stable than monomer and dimer not associated with SC [12], [36]. Consistent with this observation, our data showed that SIgA1 and SIgA2 from mAb1 and mAb2 were quite stable over the course of the experiment, with between 71% and 93% of total mAb remaining intact after two hours (Table 1). We did not observe any major differences between SIgA1 and SIgA2 for mAb1 and mAb2; however, for mAb3 and mAb4 there did appear to be a trend towards greater stability of SIgA1 as compared to SIgA2, particularly in the case of mAb4 (Fig. 3, Table2). As the two IgA subclasses are structurally distinct (with greater structural heterogeneity being found in sIgA2) [12], [18], [37], selection of one over the other may require empirical evaluation for any given application. It was notable that the protective effect of SC was much less pronounced for mAb2 and mAb4 compared to mAb1 and mAb2. Human SC contains five immunoglobulin-like domains (D1-D5), and evidence indicates that D1 is critical for the initial non-covalent association of SC with the Fc domains followed by disulfide bond formation between D5 and IgA Cα2 domain [38], [39]. Previous studies have identified heterogeneous populations of dimer of which a fraction is conformationally unable to associate properly with SC, leading to non-covalent binding [40], [41], and another study demonstrated that SC enhances stability by delaying protease cleavage within the hinge region of IgA1 and IgA2 [42]. While all mAbs tested in this assay were expressed in CHO cells, other production methods, including transgenic plants and yeast, are also being developed for production of SIgA [20], [21], [23], [43], and it is likely that structural differences due to subclass and allotype (IgA1, IgA2m1 and IgAm2) as well as the expression system and other factors may all contribute to the gastrointestinal stability of a given mAb.

Despite being cloned into the same expression vectors, the intestinal stability profiles of the four mAbs displayed considerable variation. The differences in stability observed did not appear to correlate with expression method (transient vs. stable), as stably expressed mAb3 and mAb4 SIgA1 displayed no differences in stability when compared to the transiently-expressed mAbs used in this study (data not shown). Similarly, column purification methods (CaptureSelect vs. Capto L) also did not correlate with the observed patterns. Loss of immunoreactivity in our assay could arise either from denaturation of the variable domain or complimentary determining regions (CDRs) or alternatively as a consequence of differing susceptibility to proteolytic cleavage and degradation. While the first mechanism likely explains some of the differences we observed, our results are consistent with previous studies showing that resistance to proteolytic cleavage is a primary determinant of antibody stability [35], [11], [12], [36], and suggests a possible role for variable domains in mediating stability through limiting the exposure of cleavage sites or other mechanisms. While this study did not investigate the underlying mechanisms responsible for the differences we observed between antibodies, the impact of the variable regions on stability and other molecular properties has been described in other contexts [44], [45]. A recent report found that the degree of affinity maturation of human antibodies is inversely related to thermal stability [46], and the influence of CDRs on the stability of humanized Fab displaying mouse complementarity determining regions has been also been examined [47]. Given the limited number of mAbs tested in this study we did not observe any correlation between stability and variable region features such as CDR length, divergence from germline, or gene family (Table S1), and factor(s) that may determine the impact of the variable region on proteolytic stability in the GI environment will require further evaluation.

Recombinant SIgA has been shown to display enhanced breadth against influenza virus and to protect against *Salmonellosis* via passive immunization in mouse models, and SIgA-based camelid antibody fusions can protect piglets against ETEC when delivered orally in feed [17], [18], [20], [21]. In our functional study of anti-ETEC mAbs, SIgA1 was more potent both in terms of IC_100_ and functional stability over time compared to SIgA2, particularly for mAb4, and it is possible that the presence of SC combined with the greater reach and flexibility provided by the hinge of IgA1 compared to IgA2 may explain the observed trends. As Fab fragments would also be expected to be present in these samples, our data also suggests that Fabs generated by cleavage may not be as effective as the intact antibody in binding and agglutinating bacteria, at least in this assay. In addition, and unlike full length SC associated IgA, cleaved Fabs would not be tethered to the mucous and would be more susceptible to clearance from the intestinal surface. Lastly, it is also important to note that, although mAb3 and mAb4 had poor stability profiles overall compared to mAb1 and mAb2, they did inhibit agglutination, and in the case of mAb4 SIgA1 this was true even after two hours. Thus, while any particular assay may offer insights useful for the selection of lead mAbs, final selection will need to be based upon an optimized balance of physical and immunological qualities.

In conclusion, we have used an *in vitro* assay to evaluate the proteolytic and functional stability of antibodies in the intestinal environment. Our results show that intestinal stability is influenced by isotype, subclass, variable region, and molecular structure, and highlight the utility of early stage *in vitro* assays such as the one currently described. The use of IgA-based approaches is supported by the enhanced gastrointestinal stability of the IgA isotype as compared to IgG across the mAbs tested here. Finally, these data provide additional evidence in support of the ongoing development of SIgA-based therapeutic approaches targeting enteric pathogens and disease.

## Declaration of Competing Interest

The authors declare that they have no known competing financial interests or personal relationships that could have appeared to influence the work reported in this paper.
