# Supplementary material for: IgA as a potential candidate for enteric monoclonal antibody therapeutics with improved gastrointestinal stability
Source: Vaccine. 2020 Nov 3;38(47):7490–7. doi: 10.1016/j.vaccine.2020.09.070 (PMC7604562; doi:10.1016/j.vaccine.2020.09.070)
Supplement: Supplementary data 1 [file mmc1.pptx]

## Slide 1
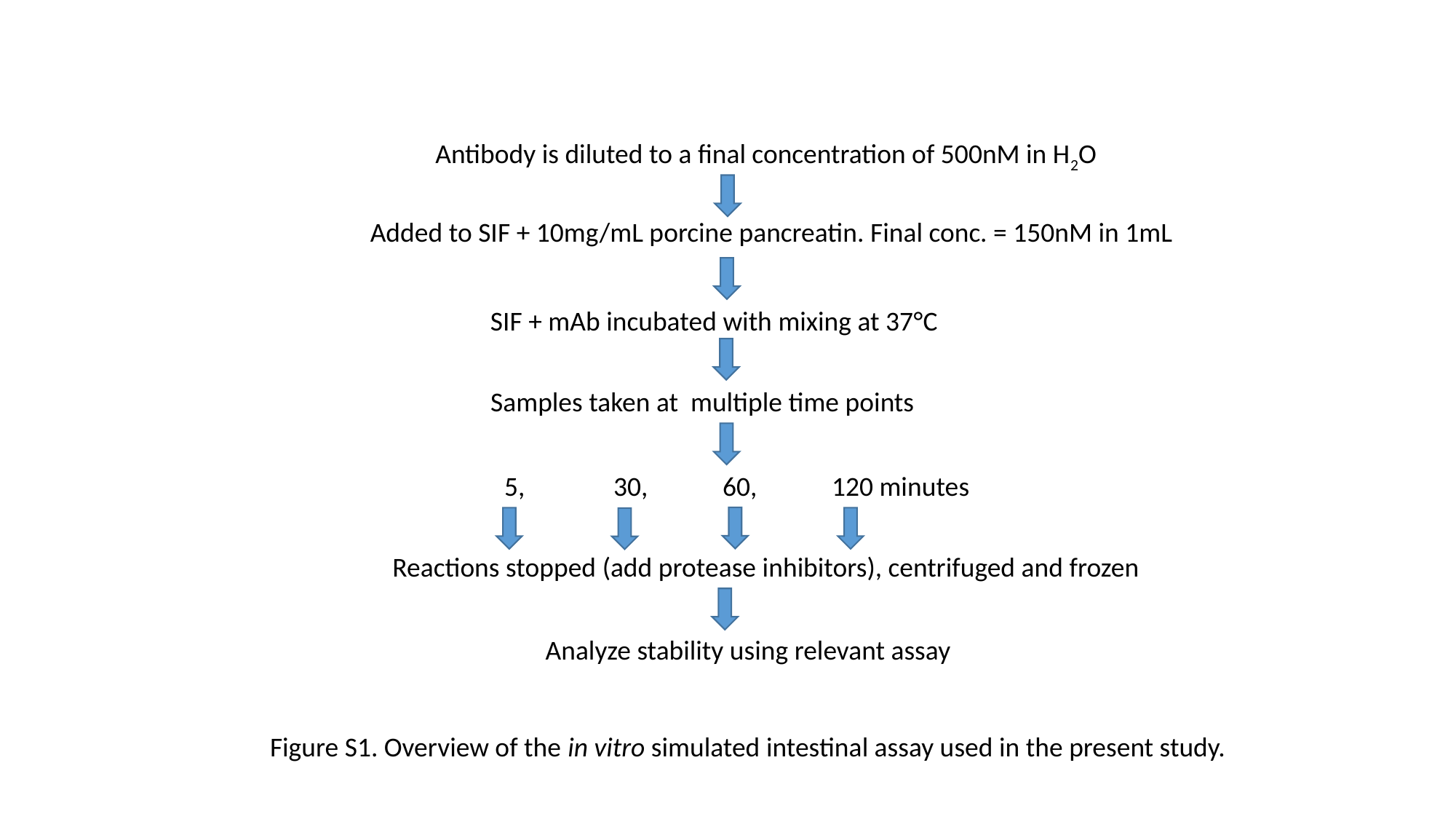

Antibody is diluted to a final concentration of 500nM in H2O
Added to SIF + 10mg/mL porcine pancreatin. Final conc. = 150nM in 1mL
SIF + mAb incubated with mixing at 37°C
Samples taken at multiple time points
5,	30,	60,	120 minutes
Reactions stopped (add protease inhibitors), centrifuged and frozen
Analyze stability using relevant assay
Figure S1. Overview of the in vitro simulated intestinal assay used in the present study.

## Slide 2
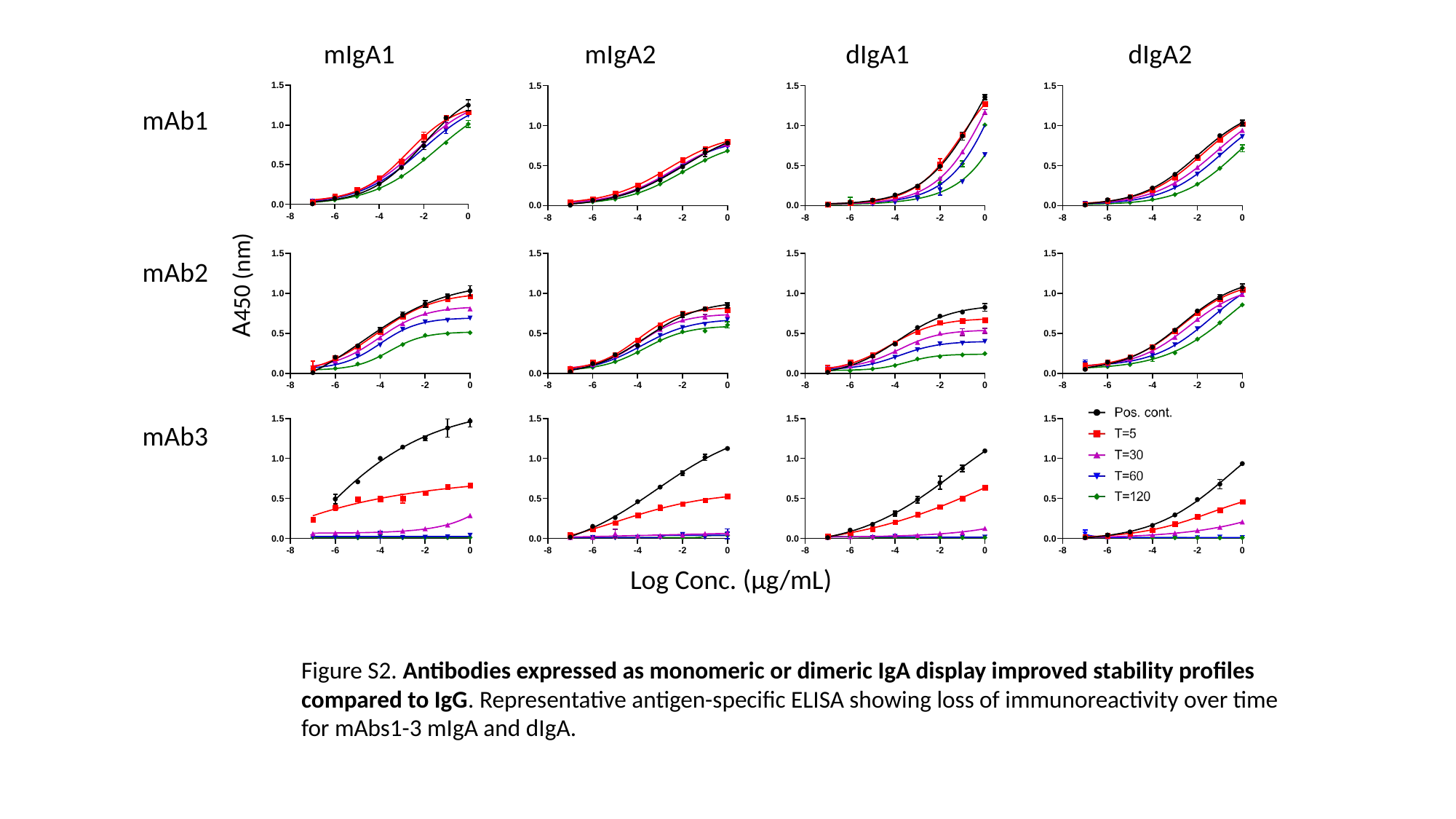

mIgA1
mIgA2
dIgA1
dIgA2
mAb1
mAb2
A450 (nm)
mAb3
Log Conc. (μg/mL)
Figure S2. Antibodies expressed as monomeric or dimeric IgA display improved stability profiles compared to IgG. Representative antigen-specific ELISA showing loss of immunoreactivity over time for mAbs1-3 mIgA and dIgA.

## Slide 3
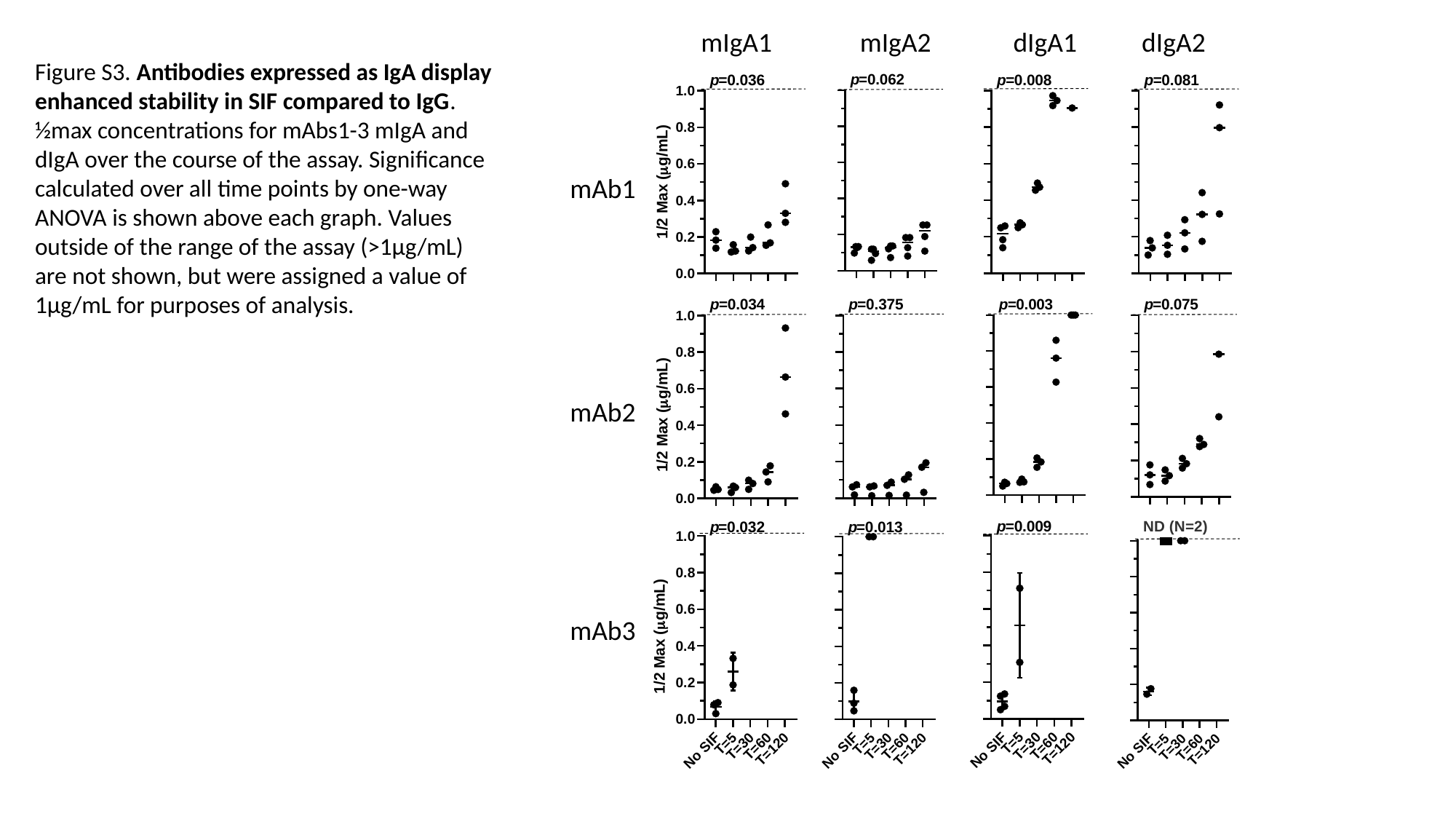

mIgA1
mIgA2
dIgA1
dIgA2
Figure S3. Antibodies expressed as IgA display enhanced stability in SIF compared to IgG. ½max concentrations for mAbs1-3 mIgA and dIgA over the course of the assay. Significance calculated over all time points by one-way ANOVA is shown above each graph. Values outside of the range of the assay (>1μg/mL) are not shown, but were assigned a value of 1μg/mL for purposes of analysis.
mAb1
mAb2
mAb3
