# Supplementary material for: IgA as a potential candidate for enteric monoclonal antibody therapeutics with improved gastrointestinal stability
Source: Vaccine. 2020 Nov 3;38(47):7490–7. doi: 10.1016/j.vaccine.2020.09.070 (PMC7604562; doi:10.1016/j.vaccine.2020.09.070)
Supplement: Supplementary data 2 [file mmc2.docx]

Table S1. **Gene family usage, % identity, and number of amino acid residues comprising each complementary determining region (CDR) for all antibodies**. Sequences were analyzed using the ImMunoGeneTics (IMGT) database with IMGT/V-Quest.

|  | **Gene Family** | **Identity (%)** | **CDR Length (amino acid residues)** | | |
| --- | --- | --- | --- | --- | --- |
|  |  |  | **CDR1** | **CDR2** | **CDR3** |
| mAb1 IGVH | IGHV4-4*02 | 99.44% | 9 | 7 | 18 |
|  | IGHJ5*02 | 94.12% |  |  |  |
|  | IGHD5-24*01 |  |  |  |  |
| mAb1 IGVK | IGKV1-9*01 | 96.06% | 6 | 3 | 9 |
|  | IGKJ1*01 | 100.00% |  |  |  |
| mAb2 IGVH | IGHV3-33*01 or *06 | 91.67% | 8 | 8 | 16 |
|  | IGHJ3*01 | 96.00% |  |  |  |
|  | IGHD3-10*01 |  |  |  |  |
| mAb2 IGVK | IGKV3-11*01 | 98.57% | 6 | 3 | 11 |
|  | IGKJ3*01 | 94.74% |  |  |  |
| mAb3 IGVH | IGHV1-69*04 | 94.79% | 8 | 8 | 12 |
|  | IGHJ3*02 | 90.00% |  |  |  |
|  | IGHD2-21*01 |  |  |  |  |
| mAb3 IGVK | IGKV1D-16*01 | 99.64% | 6 | 3 | 9 |
|  | IGKJ*01 | 100.00% |  |  |  |
| mAb4 IGVH | IGHV4-34*01 | 97.54% | 8 | 7 | 11 |
|  | IGHJ3*02 | 94.00% |  |  |  |
|  | IGHD7-27*01 |  |  |  |  |
| mAb4 IGVK | IGKV1-12*01 or *02 | 98.85% | 6 | 3 | 9 |
|  | IGKJ2*01 | 100% |  |  |  |
